# Supplementary material for: Local Accumulation of Axonal Mitochondria in the Optic Nerve Glial Lamina Precedes Myelination
Source: Front Neuroanat. 2021 May 20;15:678501. doi: 10.3389/fnana.2021.678501 (PMC8173055; doi:10.3389/fnana.2021.678501)
Supplement: Supplementary file 1 [file Data_Sheet_1.PDF]

Supplementary Material

1 Supplementary Data

Supplementary Movie S1. LSFM 3D reconstruction of an adult STOP$^{f/f}$-mitoEGFP; Vglut2-Cre (mitoRGC) optic nerve. Approximatively 0.5 mm of the nerve is shown. In the first frame, the optic nerve head is on the left part of the nerve. The tissue was labelled with an antibody against GFP.

Supplementary Movie S2. LSFM 3D reconstruction of a P8 STOP$^{f/f}$-mitoEGFP; Vglut2-Cre (mitoRGC) optic nerve. Approximatively 0.5 mm of the nerve is shown. In the first frame, the optic nerve head is on the left part of the nerve. The tissue was labelled with an antibody against GFP.

Supplementary Movie S3. LSFM 3D reconstruction of a P5 STOP$^{f/f}$-mitoEGFP; Vglut2-Cre (mitoRGC) optic nerve. Approximatively 0.5 mm of the nerve is shown. In the first frame, the optic nerve head is on the left part of the nerve. The tissue was labelled with an antibody against GFP.

2 Supplementary Figures

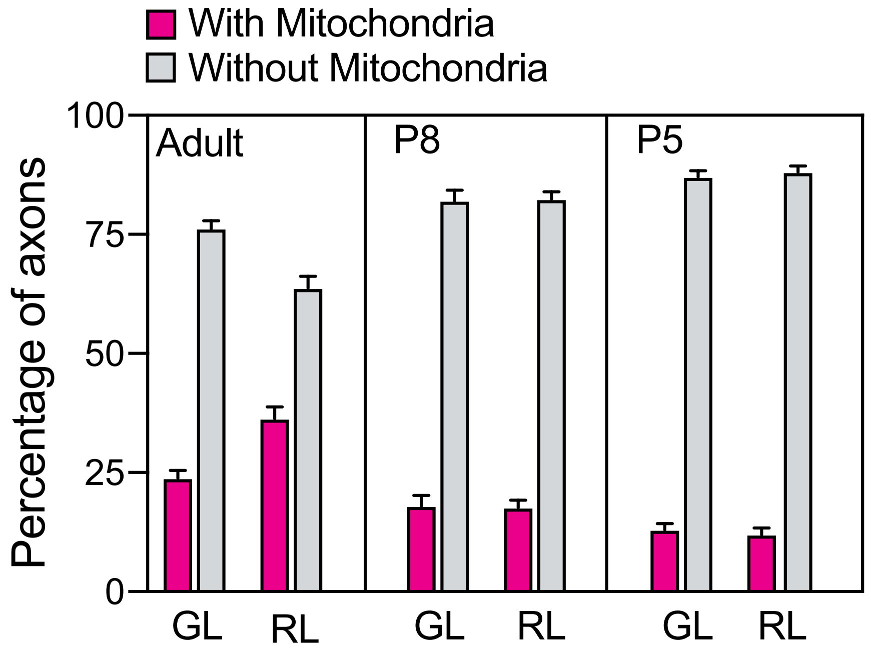

Supplementary Figure 1. The GL does not have a higher percentage of axon with mitochondria.
Percentage of axons with or without mitochondria in the GL and RL area. The number of axons with or without mitochondria was quantified on SBF-SEM single sections. Adult GL: N=3 mice, 961 axons. Adult RL: N=3 mice, 902 axons. P8 GL: N=3 mice, 1323 axons. P8 RL: N=4 mice, 1511. P5 GL: N=5 mice, 1478 axons. P5 RL: N=4 mice, 1229 axons.
